# Association of blood neutrophil lymphocyte ratio in the patients with postmenopausal osteoporosis

**DOI:** 10.12669/pjms.323.10292

**Published:** 2016

**Authors:** Chenshu Huang, Shaolin Li

**Affiliations:** 1 Dr. Chenshu Huang, PhD, MD. Department of Radiation Medicine and Tumor, College of Basic Medicine, Chongqing Medical University, Yuzhong District, Chongqing, China; 2 Prof. Shaolin Li, Department of Radiation Medicine and Tumor, College of Basic Medicine, Chongqing Medical University, Yuzhong District, Chongqing, China

**Keywords:** Neutrophil lymphocyte ratio, Postmenopausal, Osteoporosis, Bone mineral density

## Abstract

**Objective:**

To investigate the relationship between blood neutrophil lymphocyte ratio (NLR) and postmenopausal osteoporosis in Chinese women without diabetes.

**Methods:**

Two hundred and thirty three postmenopausal women without diabetes were included in this study. The clinical data of patients including age, and body mass index (BMI) were recorded. Blood samples were obtained to determine Blood urea nitrogen (BUN), Uric acid (UA), Albumin (ALB), Creatinine (CREA), Total Cholesterol(TC), Triglyceride (TG), Fasting plasma glucose (FPG), Glycosylated Hemoglobin (HbAlc), 25-hydroxyitamin D (25-OHD) level. NLR was calculated using the following formulas: NLR = Neutrophil count / lymphocyte count; Bone mineral density (BMD) of the lumbar vertebrae and the femoral neck was measured. The relationship between NLR and postmenopausal osteoporosis statistical methods was analyzed.

**Results:**

Age, BMD, Albumin (ALB), Creatinine(CREA), Triglyceride (TG) and NLR level were different in the three groups (P<0.05). Logistic regression analysis showed that the age and NLR level were risk factors for postmenopausal osteoporosis.

**Conclusions:**

NLR level was strongly associated with BMD in the postmenopausal women without diabetes, suggesting that NLR could become a helpful clinical tool in the assessment of postmenopausal osteoporosis.

## INTRODUCTION

Postmenopausal osteoporosis is a disease characterized by bone loss and microarchitectural deterioration leading to bone fragility and an increased risk of fracture.^1^ The latest data^2^ suggests that inflammation plays a critical role in bone remodeling and in pathogenesis of osteoporosis. The present study^3^ describes the neutrophil as a cell that acquires roles beyond that of a prototypic inflammatory cell, directly capable of activating osteoclasts. Blood neutrophil lymphocyte ratio (NLR) is a non-invasive, simple and cost-effective marker of inflammation in various malignancies and inflammatory diseases.^4^,^5^ Thus, the definitive link between NLR and bone mineral density(BMD) is not yet clarified and needs to be investigated. Consequently, we conducted a study of postmenopausal women without diabetes to clarify the association between NLR and both LBMD and FBMD. The objective of the present study was to compare NLR levels in osteopenic, osteoporotic and control subjects and to assess the correlation between NLR levels and BMD.

## METHODS

### Research objects

The study was carried out between April 2012 and September 2015, which was approved by the ethics committee of our center, and all participants gave their their written informed consent before enrolment into the study. We screened two hundred and thirty three postmenopausal women at our medical centers in China, who were menopausal for at least three years and were eligible for enrollment if they were 45 to 79 years old, and categorized into osteoporosis group and control group (normal BMD and osteopenia), as BMD evaluation. Those patients were excluded who had self-report diagnosis of T1D or T2D and/or they were taking antidiabetic medication and/or insulin or they had fasting plasma glucose concentration equal or more than 126 mg/dl. Those with clinical condition that affected bone metabolism, such as diseases of the liver, kidney, thyroid, or parathyroids, rheumatic diseases, malabsorption syndromes, malignant tumors, and hematological diseases were also excluded. None of those included were taking drugs or hormones that influence bone metabolism, such as glucocorticoids, estrogens, thyroid hormone, fluoride, bisphosphonate, calcitonin, thiazide diuretics, barbiturates, vitamin D, or calcium-containing drugs.

### Survey methods and quality control

Blood samples were obtained to determine Blood urea nitrogen (BUN), Uric acid (UA), Albumin (ALB), Creatinine(CREA), Total Cholesterol(TC), Triglyceride (TG), Fasting plasma glucose (FPG), Glycosylated Hemoglobin (HbAlc), 25-hydroxyitamin D (25-OHVD) level. NLR was calculated using the following formulas: NLR = Neutrophil count / lymphocyte count; Postmenopausal women underwent a BMD evaluation of the lumbar spine and left femur by DXA (Norland, XR-600, USA). BMD was expressed as the amount of mineral (g) divided by the area scanned (cm2). Bone density was then expressed as the T-score, calculated on the basis of the normal reference values. The T-score was defined as the number of standard deviations from the healthy young adult mean (normal, > 1; osteopenia, 1 to 2.49; osteoporosis, ≤ 2.5), and all cases were divided into three groups. The instrument was calibrated every day in accordance with the manufacturer’s recommendations.

### Statistical analysis

Baseline characteristics of the study subjects were summarized with means±SD for continuous variables, and by numbers or percentages for categorical items. The normality of the distribution of the study sample was assessed by Kolmogorov-Smirnov test. The presence of group differences at baseline was assessed by Student’s t test or Pearson’s 2 test for continuous and categorical items respectively. Multivariate logistic regression analysis were used to test the association between biochemical parameters and BMD in postmenopausal patients without diabetes. All statistical tests were performed by SPSS 18.0 (SPSS Inc., USA); a 2-sided P< 0.05 indicated statistical significance.

## RESULTS

The subjects were all postmenopausal for at least three years since menopause. The baseline characteristics of patients are shown in Table-I, and there were significant differences in BMI, ALB, TG, NLR, 25-OHVD and age in the three groups (p<0.05). The multivariate logistic regression analysis, the correlation between osteoporosis and BMI which disappeared is shown in Table-II. In addition, ALB, TG and 25-OHVD were not correlated. Logistic regression analysis showed that Years and NLR level were risk factors for osteoporosis (p<0.05).

**Table-I:** Comparison of baseline characteristic and biochemical parameters in three groups.

*Variable*	*Normal group (n=51)*	*Osteopenia group (n=60)*	*Osteoporosis group (n=122)*	*F*	*P*
Age(year)	50.55±9.18	56.09±9.79	67.07±9.66	8.803	0.000
BMI(kg/m2)	23.80±2.71	23.35±2.48	20.21±3.09	16.082	0.000
BUN(mmol/L)	6.23±1.57	6.35±1.72	6.47±1.83	0.212	0.846
UA(μmol/L)	332.05±83.37	352.63±89.99	343.38±88.73	0.606	0.553
ALB(g/L)	39.95±4.74	39.47±4.64	36.03±3.21	6.615	0.002
CREA(μmol/L)	79.02±14.75	80.00±12.68	73.24±11.48	1.629	0.211
TC(mmol/L)	3.91±0.82	3.83±0.83	3.47±0.89	2.790	0.065
TG(mmol/L)	1.32±0.57	1.15±0.53	1.09±0.58	4.037	0.021
FPG(mmol/L)	4.93±1.15	5.18±0.97	5.34±1.03	1.618	0.202
HbAlc(%)	6.15±0.61	6.12±0.52	6.11±0.50	0.149	0.862
NLR	2.12±0.89	2.55±1.15	2.74±1.06		
25-OHVD(ng/ml)	15.68±6.95	13.26±5.95	10.59±5.55		

**Table-II:** Multivariate logistic regression analysis results of the possible factors of osteoporosis in patients without diabetes.

	*OR*	*95%*	*P*
Age	1.051	1.040 ~ 1.080	<0.001
BMI	0.901	0.769 ~ 1.061	0.212
NLR	1.018	0.941-1.101	0.034
25-OHD	0.946	0.888-1.008	0.084
ALB	0.939	0.860-1.042	0.251
TG	0.568	0.222-1.441	0.232

## DISCUSSION

The results of this study in Chinese postmenopausal females not affected by diabetes showed that NLR level was higher in the postmenopausal osteoporosis group (P<0.05). Multivariate Logistic regression analysis showed that the NLR may be a risk factor for postmenopausal osteoporosis. With these results, we inferred that NLR could be a novel osteopenic marker in postmenopausal women not affected by diabetes, if confirmed.

Increasing evidence seemed to indicate that the host inflammatory response was correlated with the occurrence and development of OP, so that it could serve to predict the clinical outcomes of patients with OP. Several studies^6^ had shown that an elevated NLR was associated with poor prognosis in patients with OP.

Number of studies^7^ have suggested that there was a close relationship between osteoporosis and inflammatory diseases, such as rheumatoid arthritis, inflammatory bowel disease, systemic lupus erythematosus, chronic obstructive pulmonary disease. In these diseases, inflammation on the one hand led to the generation of the primary disease, on the other hand could affect the skeletal system, disturbances of bone metabolism balance.in vitro and animal experiments^8^ found that there was a significant correlation between BMD and inflammatory factors, such as IL1, IL-6, CRP, TNF-α. Another study^9^ showed that older women with high levels of inflammatory cytokines had increased risk of hip fractures, which suggested the importance of inflammation in osteoporosis and the effect of inflammatory cytokines on bone metabolism was mainly through RANK/RANKL/OPG pathway. In recent years, there were more and more studies of classification and counting of WBC, which suggested that NLR was closely associated with inflammatory disease, cardiovascular disease, colon cancer and lung cancer, and had also shown that NLR could predict adverse outcomes in patients with cardiovascular disease and cancer.^10^,^11^

Poubelle found^12^ that in certain inflammatory conditions, neutrophils would be directly involved in acquired immunity or in bone remodeling through their expression of RANK, depending on the factors present simultaneously at the inflammatory site. Chakravarti reported^13^ that inflammatory bone loss in septic and inflammatory conditions was due to increased activity of osteoclasts that required receptor activator of NF-kappa B-ligand (RANKL), while neutrophils were the predominant infiltrating cells in these conditions. Oztirk reported^14^ that elderly people with osteoporosis had elevated NLR levels, suggesting that inflammation could play an important role in bone remodeling.

### Limitations of the Study

This study had small sample size and cross-sectional design, which could not determine causality. Furthermore, the investigation was done on representative samples of the Chinese population.

The results of this study in Chinese postmenopausal females not affected by diabetes showed that NLR level may be a risk factor for osteoporosis and further studies were warranted to investigate the potential relationship between NLR and bone loss in other population and to confirm that NLR could become a helpful clinical tool in the assessment of postmenopausal osteoporosis.
